# Non-operative Management of a Diaphragmatic Hematoma: A Case Report

**DOI:** 10.7759/cureus.80484

**Published:** 2025-03-12

**Authors:** Taylor M Gong, Kendall Ozorowski, Davin J Evanson, Michael A Romeo

**Affiliations:** 1 Department of Radiology, Drexel University College of Medicine, Wyomissing, USA; 2 Department of Radiology, Sidney Kimmel College of Medicine, Thomas Jefferson University, Philadelphia, USA; 3 Division of Diagnostic Radiology, Reading Hospital, West Reading, USA

**Keywords:** blunt injury, computed tomography (ct) imaging, diagnostic imaging, hematoma, motor vehicle accident, non-operative management, traumatic diaphragmatic injury

## Abstract

Diaphragmatic hematomas are a rare complication of blunt trauma that are often overlooked due to the presence of high-acuity injuries. However, this injury can result in significant long-term complications if not managed appropriately. We present a 59-year-old female who developed a diaphragmatic crural hematoma following a motor vehicle accident. The patient arrived at the trauma bay unresponsive, hypertensive, requiring bag-valve mask ventilation, and with a Glasgow Coma Scale score of 3/15. On physical examination, the patient was found to have bilateral diminished breath sounds and was promptly intubated. Initial CT imaging of the patient demonstrated active contrast extravasation that was thought to be an adrenal hemorrhage but was actually a diaphragmatic hematoma upon re-examination. Although surgical intervention is a common approach for managing diaphragmatic injuries, non-operative management was chosen in this case due to the patient's stable condition and minimal evidence of active bleeding. This case highlights the possibility of non-operative management of diaphragmatic hematomas when used with accurate radiologic and clinical findings. When appropriate, non-operative management can reduce morbidity and mortality but requires careful radiologic follow-up to detect potential complications.

## Introduction

Acute traumatic diaphragmatic injury (TDI) is a rare result of major blunt trauma, occurring in less than 10% of trauma patients [1]. The most frequent causes are motor vehicle accidents and falls from significant heights [2]. Due to the acute nature of the etiology, diagnosis of a TDI poses a significant challenge, as other life-threatening thoracic and/or intra-abdominal injuries warrant immediate attention and may obscure its detection [2]. Undiagnosed TDI can lead to long-term complications, including increased morbidity and mortality, often presenting later as symptomatic herniation of viscera, which requires surgical repair [3,4]. In both hemodynamically unstable and stable patients, TDIs are often treated and repaired with exploratory laparotomy as they are difficult to identify radiologically [5]. To our knowledge, non-operative management of a diaphragmatic crural hematoma without additional intra-abdominal lesions has been described in only one other case report [6]. We report a rare case of non-operative management of a diaphragmatic hematoma following blunt trauma in a 59-year-old female.

## Case presentation

**Figure 1:**
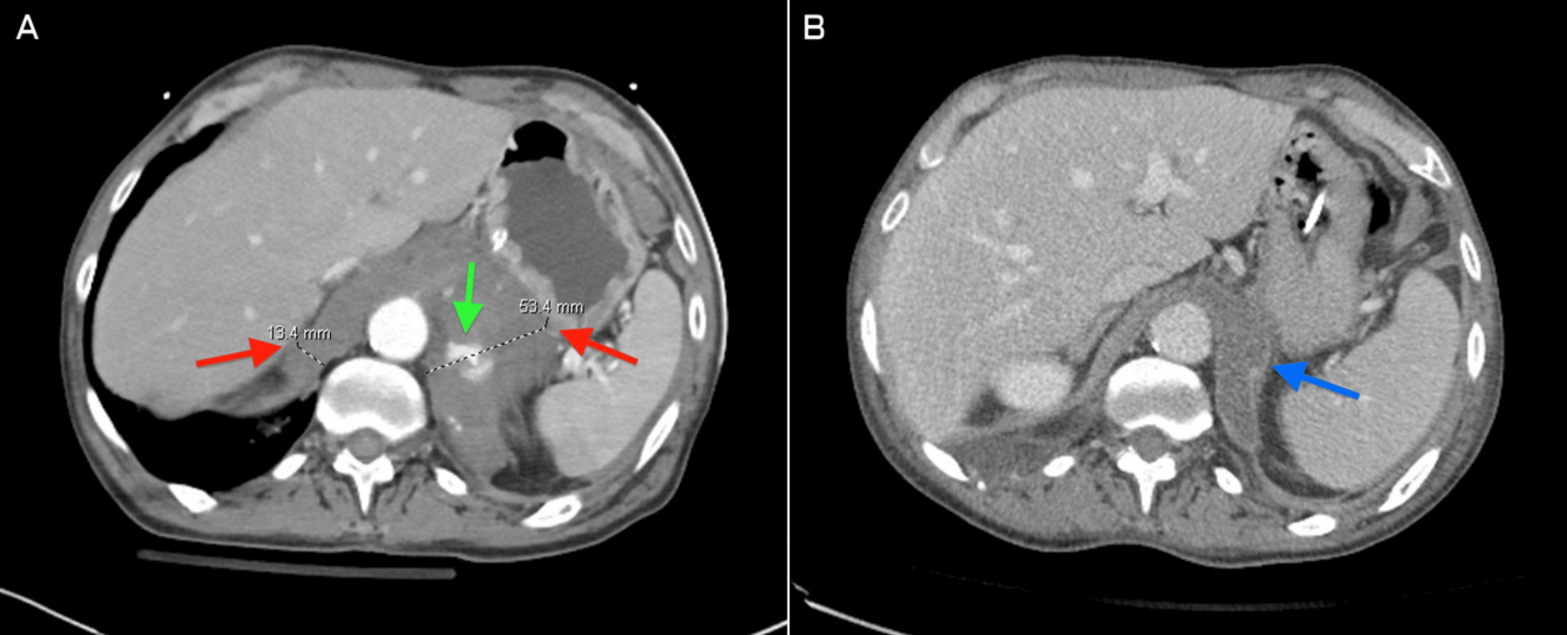
(A) Initial abdominal axial CT demonstrating active contrast extravasation (green arrow) and heterogenous thickening of the right and left diaphragmatic crura, measuring 13.4 mm and 53.4 mm, respectively (red arrows), consistent with hematoma within the right and left diaphragmatic crura. (B) 15-day follow-up. Abdominal axial contrast-enhanced CT shows near-complete resolution of the right diaphragmatic hematoma and significant reduction in size of the left diaphragmatic hematoma (blue arrow). CT: computed tomography

A 59-year-old female presented to the emergency department following a pedestrian vs. motor vehicle accident. The patient was found unresponsive 20 feet from the vehicle, which had been traveling at approximately 30-40 mph. Following arrival to the trauma bay, the patient had a Glasgow Coma Scale score of 3/15 and was hypertensive, with ventilation being assisted by a bag-valve mask. During the physical examination, the patient was found to have bilateral diminished breath sounds. The patient was immediately intubated, and a CT scan with contrast was obtained. The CT scan showed left lower lobe pulmonary contusions, left L3-L5 transverse process fractures, a bilateral comminuted sacral fracture, and an area of active extravasation anterolateral to the T12/L1 vertebrae. The source of extravasation was unclear, and a left adrenal hemorrhage was suspected (Figure 1A).

**Figure 2:**
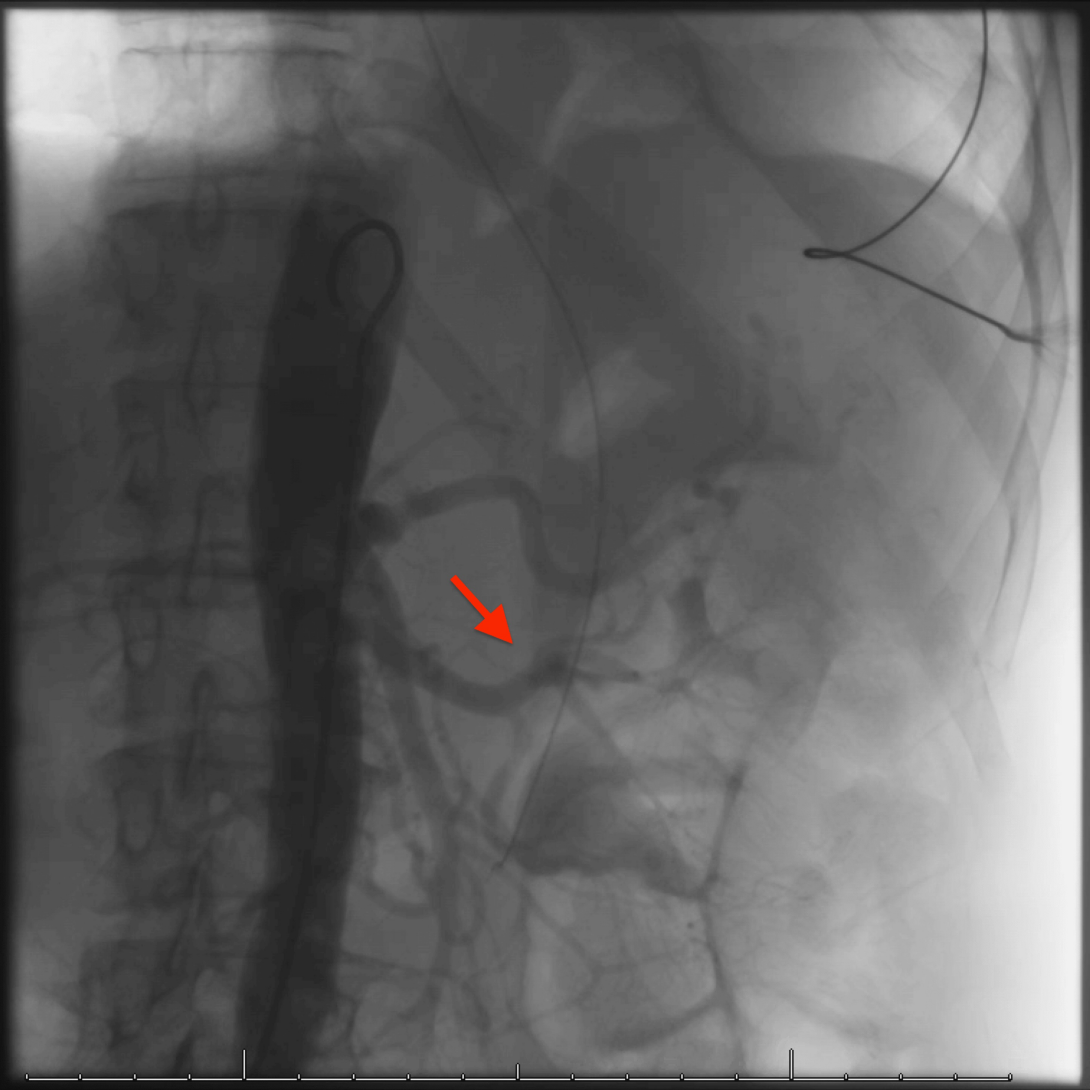
Abdominal aortogram demonstrating intact left renal and adrenal arteries with no active extravasation visualized (red arrow)

To identify the source of hemorrhage, an abdominal aortogram was performed; however, contrast flow showed no abnormalities of adrenal vasculature (Figure 2).

Following this procedure, there was concern that the left adrenal hemorrhage was actually a left diaphragmatic crus hematoma with active extravasation, which was later confirmed on the 15-day follow-up CT (Figure 1B). The patient was monitored for signs of respiratory failure and internal bleeding; however, the patient remained hemodynamically stable, and the diaphragmatic hematoma was managed non-operatively. The patient’s hospital course focused on pain management, and on day 23 post-admission, the patient was discharged to a skilled nursing facility.

## Discussion

TDIs associated with blunt trauma commonly result from motor vehicle accidents and direct impact to the abdomen, frequently leading to more obvious injuries that mask a TDI. However, these injuries often carry direct and indirect signs that should raise a high suspicion index for possible diaphragm involvement. Direct signs of TDI include herniation of abdominal viscera into the thoracic cavity and the dependent viscera sign, where herniated viscera rest against the posterior thoracic wall due to loss of diaphragmatic support. Indirect signs include elevated hemidiaphragm, high injury severity scores, associated injuries, and contrast extravasation at the level of the diaphragm [2,7].

Our patient presented with multiple injuries as a result of blunt trauma, including pulmonary contusions, sacral and lumbar fractures, and active contrast extravasation at the level of the diaphragm (Figure 1A). Although our patient did not have any direct signs of a diaphragmatic injury, the combination of these indirect signs and presenting etiology should have created a moderate index of suspicion for possible diaphragmatic involvement [8,9]. A CT scan is considered the gold standard for TDI, with meta-analysis showing high sensitivity and specificity at 80% and 98%, respectively, while X-ray has low sensitivity [10]. Distinct imaging signs of diaphragmatic rupture have been described, being placed into categories of direct signs of rupture (e.g., diaphragmatic discontinuity), indirect signs that are consequences of rupture (e.g., intrathoracic herniation of viscera), and uncertain signs of origin (e.g., peri-diaphragmatic injury) [8,9]. Enlargement of the diaphragm has been a well-studied indication of diaphragmatic rupture, with one study finding high specificity with an average thickness of 5.75 mm in blunt trauma patients [11]. However, abnormal enlargement, such as in our case of 53.4 mm in the left diaphragmatic crus (Figure 1A), may indicate a diaphragmatic hematoma. However, more sample data will need to be obtained to support this hypothesis and determine the value at which this distinction can be made.

Blunt diaphragmatic injury is usually treated with exploratory laparotomy, as they are difficult to identify and repair. However, this patient’s management was non-operative due to minimal contrast extravasation on radiographic imaging, suggesting hemorrhage resolution (Figure 1B). Had the patient been hemodynamically unstable, an exploratory laparotomy may have been warranted. However, this case highlights the difficulty in solely relying on radiographic diagnosis, as an adrenal hemorrhage was the initial suspicion of internal injury.

TDIs of clinical significance that present as hernia of viscera have long been observed, studied, and recognized as causes of increased mortality. Still, cases of diaphragmatic hemorrhage have rarely been reported [7,12,13]. Repeat imaging is suggested at 6-to-12-month intervals after occurrence to identify or rule out a missed diaphragmatic injury [2,14]. However, one limitation of this report is that there was no long-term follow-up imaging to assess for complications or determine if operative management was warranted. Misdiagnosis or lack of diagnosis can increase morbidity and mortality. Therefore, identification, appropriate treatment, and follow-up are paramount.

## Conclusions

Crural diaphragmatic hematoma is a rare, difficult-to-diagnose complication of major blunt trauma that is not commonly reported in literature. Typically, invasive exploratory laparotomy is performed for suspected diaphragmatic hemorrhage to identify and embolize the source of hemorrhage. However, this case portrays successful non-operative management. Initial CT imaging of the patient demonstrated active contrast extravasation that was mistakenly diagnosed as an adrenal hemorrhage. The patient was hemodynamically stable, and the contrast extravasation was minimal, allowing for non-operative management. When appropriate, non-operative management can reduce morbidity and mortality, but it requires careful radiologic follow-up to detect potential complications, including herniation of viscera.
